# MRI-detected spinal disc degenerative changes in athletes participating in the Rio de Janeiro 2016 Summer Olympics games

**DOI:** 10.1186/s12891-020-3057-3

**Published:** 2020-01-20

**Authors:** Mohamad Abdalkader, Ali Guermazi, Lars Engebretsen, Frank W. Roemer, Mohamed Jarraya, Daichi Hayashi, Michel D. Crema, Asim Z. Mian

**Affiliations:** 1 Department of Radiology, Boston University School of Medicine, 820 Harrison Avenue, FGH Building 3rd Floor, Boston, MA 02118 USA; 2 Medical and Scientific Department, International Olympic Committee, Lausanne, Switzerland; 3 Oslo Sports Trauma Research Center, Department of Sports Medicine, Norwegian School of Sport Sciences, Oslo, Norway; 4 Department of Orthopedic Surgery, Oslo University Hospital, University of Oslo, Oslo, Norway; 5 Department of Radiology, University of Erlangen-Nuremberg, Erlangen, Germany; 6 Department of Radiology, Brigham and Woman Hospital, Boston, MA USA; 7 Department of Radiology, Stony Brook Medicine, Stony Brook, NY USA; 8 Institute of Sports Imaging, French National Institute of Sports (INSEP), Paris, France

**Keywords:** Degenerative disc disease, Olympic games, Sports-related injury, Spine imaging, MRI

## Abstract

**Objective:**

To describe the frequency and the distribution of degenerative disc disease (DDD) detected in athletes who underwent spine MRI in the 2016 Summer Olympic Games in Rio de Janeiro.

**Methods:**

Data on spine MRI examinations from the 2016 Summer Olympics were retrospectively analyzed. We assessed the frequency of DDD of the cervical (Cs), thoracic (Ts), and lumbar (Ls) spine using Pfirrmann’s classification. Grade II and III were considered as mild, grade IV as moderate, and grade V as severe disc degeneration. Data were analyzed according to the location of the degenerative disc, type of sport, age-groups, and gender of the athletes.

**Results:**

One hundred out of 11,274 athletes underwent 108 spine MRI’s (21 C, 6 T, and 81 L) (53% Females (F), 47% Males (M)). The frequency of DDD was 40% (42% F, 58% M) over the entire spine (28% mild, 9% moderate and 3% severe). There were 58% (12%F, 88%M) of the cervical spine discs that showed some degree of degeneration (44% mild, 13.5% moderate and 1% severe). Athletics, Boxing, and Swimming were the sports most affected by DDD in the Cs. There were 12.5% of the thoracic discs that showed some degree of degeneration, all were mild DDD and were exclusively seen in female athletes. There were 39% (53% F, 47% M) of the lumbar discs with DDD (26% mild, 9% moderate, and 4% severe).

**Conclusion:**

Athletes who underwent spine MRI during the 2016 Summer Olympic Games show a high frequency of DDD of cervical and lumbar spines. Recognition of these conditions is important to develop training techniques that may minimize the development of degenerative pathology of the spine.

## Introduction

Several studies have shown associations between competitive sports activities and degenerative pathology of the spine [1–13] . *Olympic sports* are no exception since Olympic athletes spend a considerable amount of time training and competing relying on strength, speed, force, bending, and twisting in their sport. The high intensity and repetitiveness of these activities predispose athletes to accelerated degenerative disc disease (DDD), theoretically higher than the general population [3, 6, 7]. Disc degeneration is considered the first step of degenerative disease of the spine, and it is usually followed by intervertebral disc narrowing, osteophyte formation, and resultant spinal stenosis and may be associated with pain and other neurological symptoms [14]. The aim of our study is to assess the frequency and the distribution (by location, gender distribution, type of sport, and age groups) of the degenerative discs detected on 108 MRIs of athletes competing in 28 sports in the 2016 Summer Olympic Games in Rio de Janeiro, Brazil since recognition of these conditions may assist in developing techniques to prevent early degenerative changes in spine.

## Methods

A retrospective review was performed of patients’ database and imaging data of athletes participating in the Rio Summer Olympics.

### Confidentiality and ethical approval

Our study was approved by the International Olympic Committee (IOC) (R2C10). An institutional review board (IRB) approval was obtained from Boston University (#H-36593). Data were collected, stored and analyzed in strict compliance with data protection and athlete confidentiality. Informed consent was waived because all data was anonymized and unidentifiable.

### Data collection

All Olympic athletes were permitted to walk into the Olympic village clinic and get a spine MRI if they had pain in the neck, mid or lower back. From the data that we received, there was no information regarding pain location, pain intensity or whether the pain was from a recent injury or chronic.

MRI imaging was obtained within the Olympic village using 3 T Discovery MR750w and 1.5 T Optima 450MRw MRI scanners (GE Healthcare, Brazil). For the cervical spine, sagittal T1 and T2-weighted and short tau inversion recovery sequences and axial two-dimensional multiple echo recombined gradient echo (2D MERGE) and isotropic 3D CUBE T2-weighted were obtained. For the thoracic spine, sagittal T1 and T2-weighted and short tau inversion recovery sequences and only one axial 2D MERGE plane were obtained. For the lumbar spine, sagittal T1 and T2-weighted and short tau inversion recovery sequences and axial T1 and T2-weighted images were obtained.

### Imaging interpretation

**Fig. 1:**
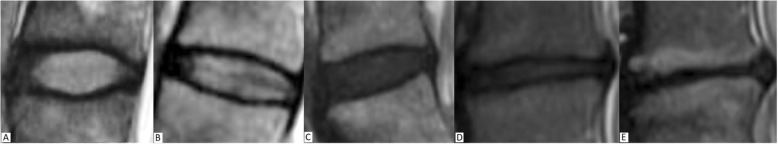
Pfirrmann grading system for disc degeneration on sagittal T2 weighted images. **a**: Grade I, bright and homogeneous disc with clear distinction between nucleus pulposus and annulus fibrosis. Normal disc height. **b**: Grade II, inhomogeneous disc with horizontal dark band. Nucleus and annulus are clearly differentiated. Preserved disc height. **c**: Grade III, dark disc with unclear distinction between nucleus and annulus. Disc height is usually normal. **d**: Grade IV, dark and heterogeneous disc with decreased disc height. **e**: Grade V, dark and collapsed disc with no distinction between the nucleus and annulus

MRI examinations were reviewed by a board-certified neuroradiologist (AZM) with 14 years of experience in spine imaging. Pfirrmann classification grading system was used to grade lumbar disc degeneration and it was extended to the evaluation of the cervical and thoracic spine [15]. In this study, a T2-weighted sequence is used to grade the disc degeneration (Fig. 1). Discs were categorized as normal (grade 1) or degenerated (grades II, III, IV and V). We considered Pfirrmann’s grades II and III as mild DDD since there was no loss of disc space height. Pfirrmann’s grade IV was considered moderate due to reduced disc space height and grade V was considered severe due to collapsed disc space.

## Results

A total of 11,274 athletes from 207 National Olympic Committees (NOCs) competed in 28 sports and 306 sporting events. One hundred participants underwent 108 MRI’s of the spine for a variety of reasons with a total of 603 intervertebral discs [16].

**Fig. 2:**
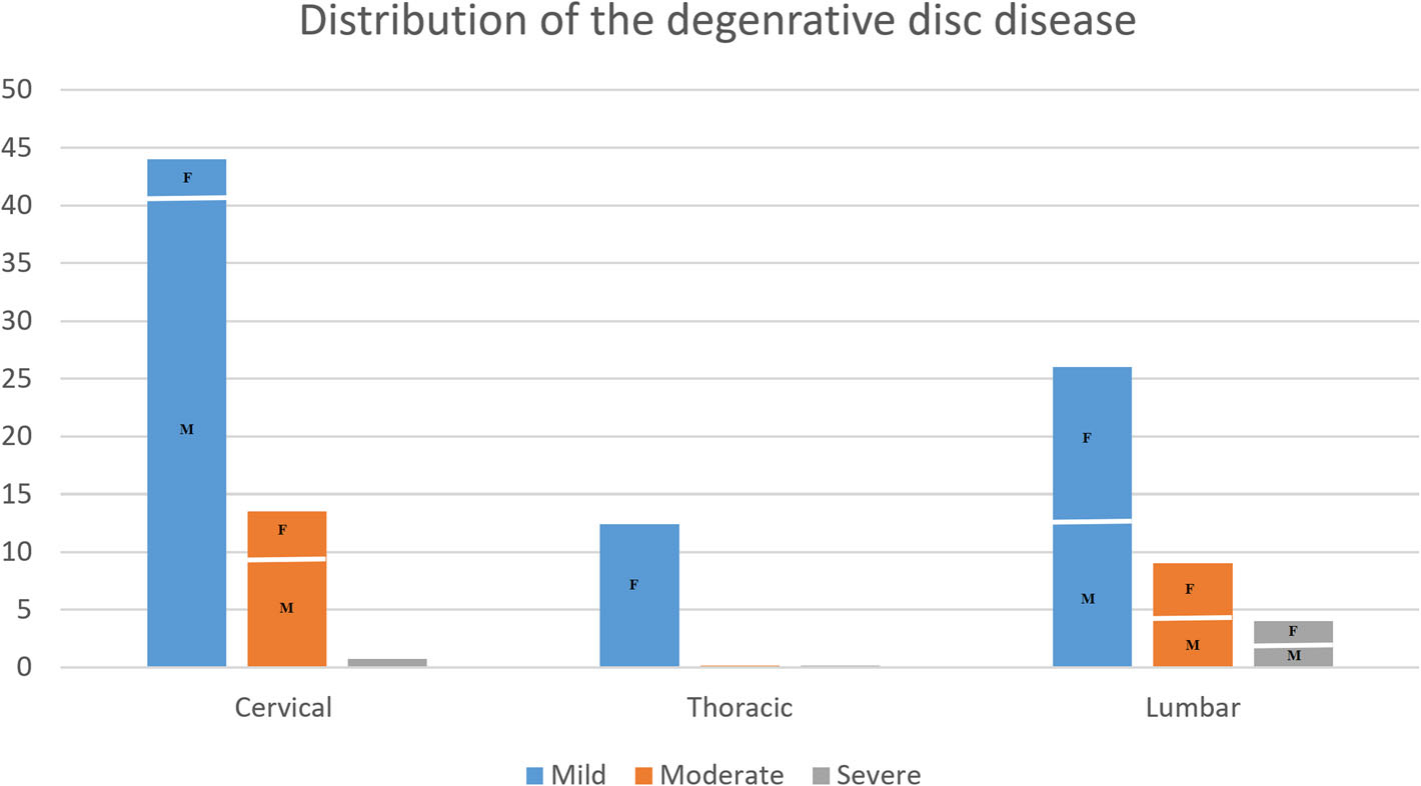
A clustered column chart showing the breakdown of the cervical, thoracic and lumbar spine MRI’s with gender distribution (F: female, M: Male)

Distribution of Pfirmanns type I-V degenerative disc disease in the cervical, thoracic and lumbar spine MRI of all athletes are showed in Table 1. Distribution and grading of the degenerative disc disease in the cervical, thoracic and lumbar spine MRI with gender distribution are showed in Table 2 and in Fig. 2 as clustered column chart. The breakdown of the subjects such as age, gender, type of sports by the level of the cervical and lumbar spine are showed in the Additional files 1 and 2. The Kappa scores for intra- and interobserver agreement of Pfirrmann disc degeneration grading were 0.8393 (95% CI = 0.7731–0.9054) and 0.8391 (95% CI = 0.7779–0.9004), respectively. For both intra- and inter-reader reliability, the kappa scores indicate a strong agreement.

**Table 1:** Distribution of Pfirmanns type I-V degenerative disc disease in cervical, thoracic and lumbar spine MRI’s of all athletes

Pfirmanns grade	Cervical	Thoracic	Lumbar	
I	53	63	247	363
II	11	1	69	81
III	44	8	36	88
IV	17	0	38	55
V	1	0	15	16
	126	72	405	603

**Table 2:** Distribution and severity grading of degenerative disc disease in cervical, thoracic and lumbar spine MRI’s with gender distribution

	Cervical	Thoracic	Lumbar
Mild	44%	12.4%	26%
	W: 7%	W: 100%	W: 50%
	M: 93%		M: 50%
Moderate	13.5%	0%	9%
	W: 30%		W: 55%
	M: 70%		M: 45%
Severe	0.7%	0%	4%
	W: 30%		W: 60%
	M: 100%		M: 40%
Total	58.2%	12.4%	39%

### Cervical spine

Twenty-one participants underwent MRI of the cervical spine with a total of 126 cervical discs. There were 53 cervical discs categorized as Grade I (42%), 11 discs as Grade II (8%), 44 discs as Grade III (35%), 17 discs as Grade IV (13.5%) and one disc as grade V (0.7%).

### Mild degenerative changes (grade II and III)

Approximately 44% or 55/126 of the cervical disks showed mild degenerative changes (Grade II and III). They were predominantly seen at the mid and lower cervical spine (C3-C4, C4-C5 and C5-C6) (82% or 45/55). Males were 10 times more affected than females (93% or 51/55 versus 7% or 4/55). Approximately 44% or 24/55 of mild degenerative changes were seen between 20 and 30 years of age and 56% (31/55) were seen above 30 years of age. Shooting (18% or 10/55) and Judo (16% or 9/55) were the sports most affected by mild DDD.

### Moderate degenerative changes (grade IV)

**Fig. 3:**
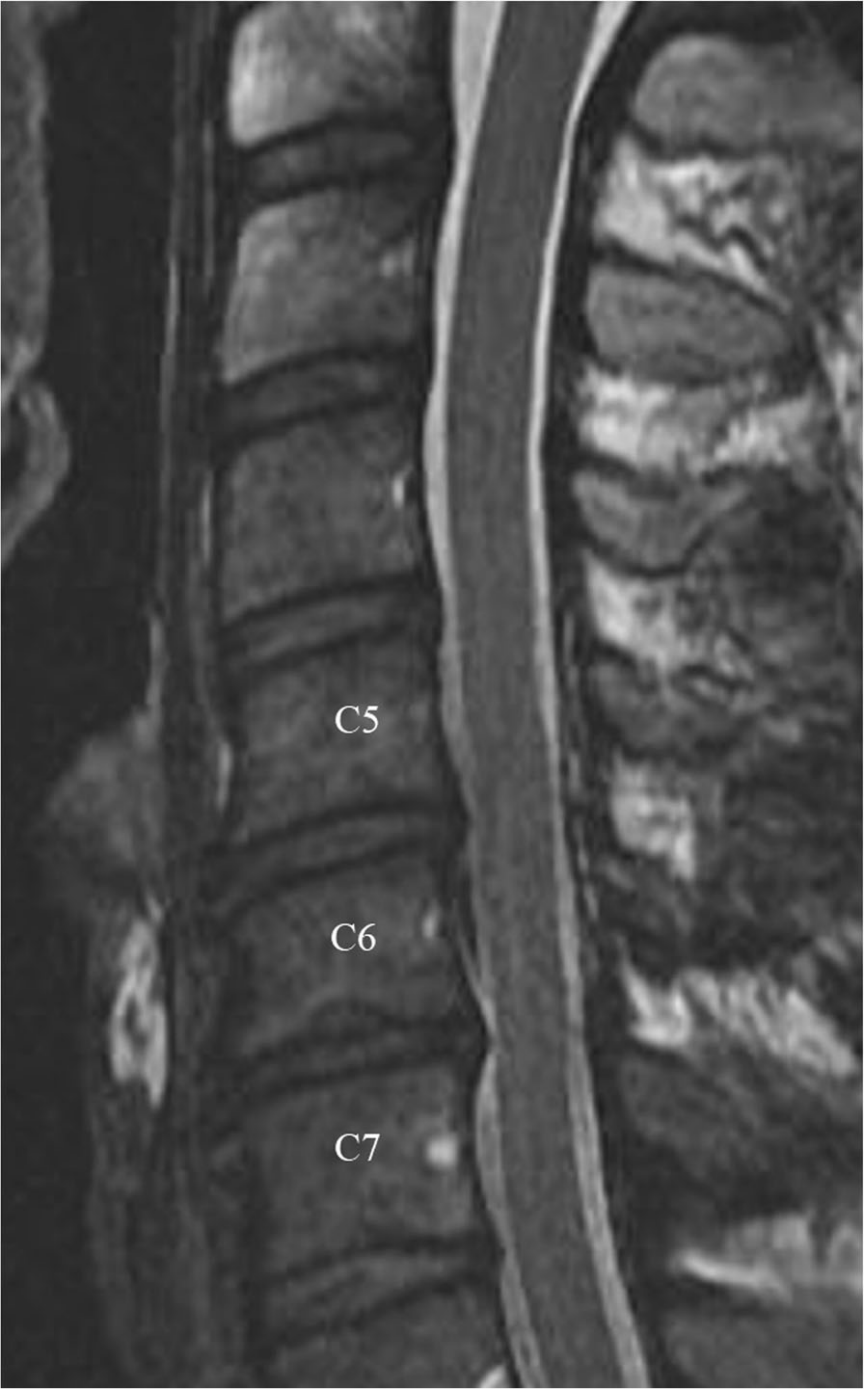
Sagittal T2 weighted images of the cervical spine of a male basketball player showing disc dissecation at C5–6 and C6–7. There is also decreased disc space with loss of the differentiation between the annulus fibrosis and nucleus pulposus at C6–7, consistent with Pfirrmann grade IV disc degeneration. Note is made of disc-osteophyte complex at C5–6 and C6–7 and lower C6 Shmorl’s node

There were 13.5% or 17/126 of the cervical disks that showed grade IV or moderate degenerative disc changes (Fig. 3). C5-C6 and C6-C7 levels were the most affected (9/17 or 53%). Nearly 70% or 12/17 were seen in males and 30% or 5/17 in females. Approximately 41% or 7/17 of the affected disks were seen between the age of 20 and 30 and 58% or 10/17 above the age of 30 years of age. Athletics (5/17 or 29%), boxing (5/17 or 29%) and swimming (3/18 or 17%) were the sports most affected by grade IV disc disease.

### Severe degenerative changes or grade V

Only one disk with grade V or severe degenerative disease was found 1/126 (0.7%). It was seen in a male sailor above 30-years of age.

#### Thoracic spine

Six participants underwent MRI of the thoracic spine with a total of 72 thoracic discs. There were 63 Grade I (87.5%), 1 Grade II (1.4%), 8 Grade III (11%), zero grade IV and zero grade V (0%). T8-T9 was the most commonly affected level by mild DDD. Gymnastics and Aquatics Diving female athletes between 20 and 30 years were the only affected participants.

#### Lumbar spine

Eighty-one participants underwent MRI of the lumbar spine with a total of 405 lumbar discs. There were 247 discs as Grade I (61%), 69 grade II (17%), 36 grade III (9%), grade IV (9%) and 15 grade V (4%).

### Mild degenerative changes or grade II and III

There were 26% or 105/405 disks with mild DDD. L4-L5 and L5-S1 levels were the most affected (47% or 49/105). Females and males were affected equally (50% or 53/105 versus 50% or 52/105). Most of the affected discs were seen between the age of 20 and 30 (51% or 54/105) versus 40% or 42/105 above 30-year-old of age and 9% or 9/105 below the age of 20. Athletics were the sports most affected by mild disc disease (29% or 30/105) followed by weightlifting (10% or 10/105) and Judo (10% or 10/105).

### Moderate degenerative changes or grade IV

**Fig. 4:**
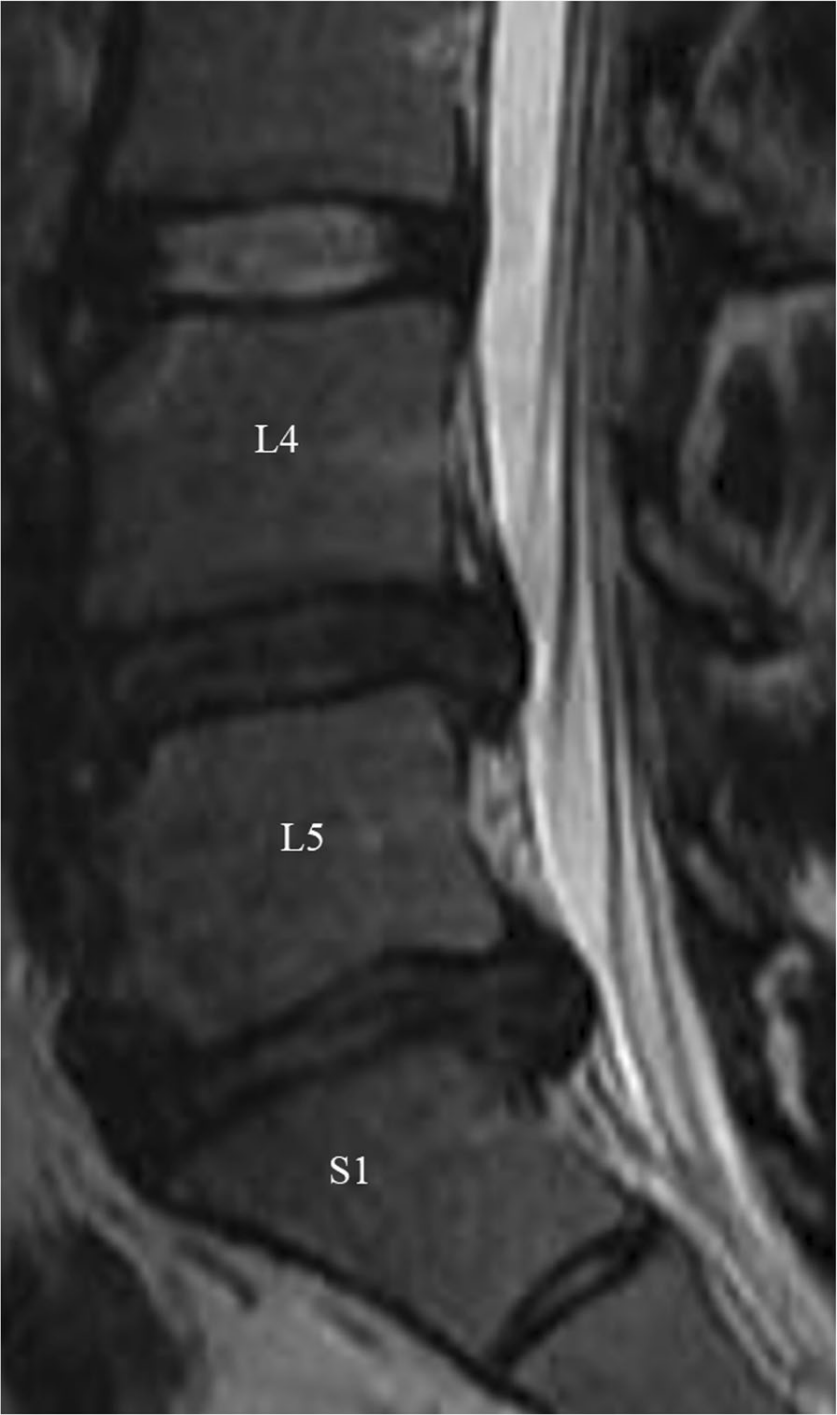
Sagittal T2 weighted images of the lumbar spine of a female sailor. There is a heterogeneous appearance with disc dissecation at L4–5 and L5-S1 with decreased disc space and disc extrusion at L5-S1, findings are consistent with Pfirrmann grade IV and V, respectively

There were 9% or 38/406 disks with grade IV or moderate DDD (Fig. 4). L4-L5 and L5-S1 levels were the most affected (82% or 31/38). Females were slightly more commonly affected than males (55% or 21/38 versus 45% or 17/38). The affected disks were equally seen between age of 20 and 30 (50% or 19/38) and above age of 30 (50% or 18/38). Athletics were the sports most affected by grade IV moderate disc disease (32% or 12/38) followed by Diving (13% or 5/38) and weightlifting (10% or 4/36).

### Severe degenerative changes or grade V

**Fig. 5:**
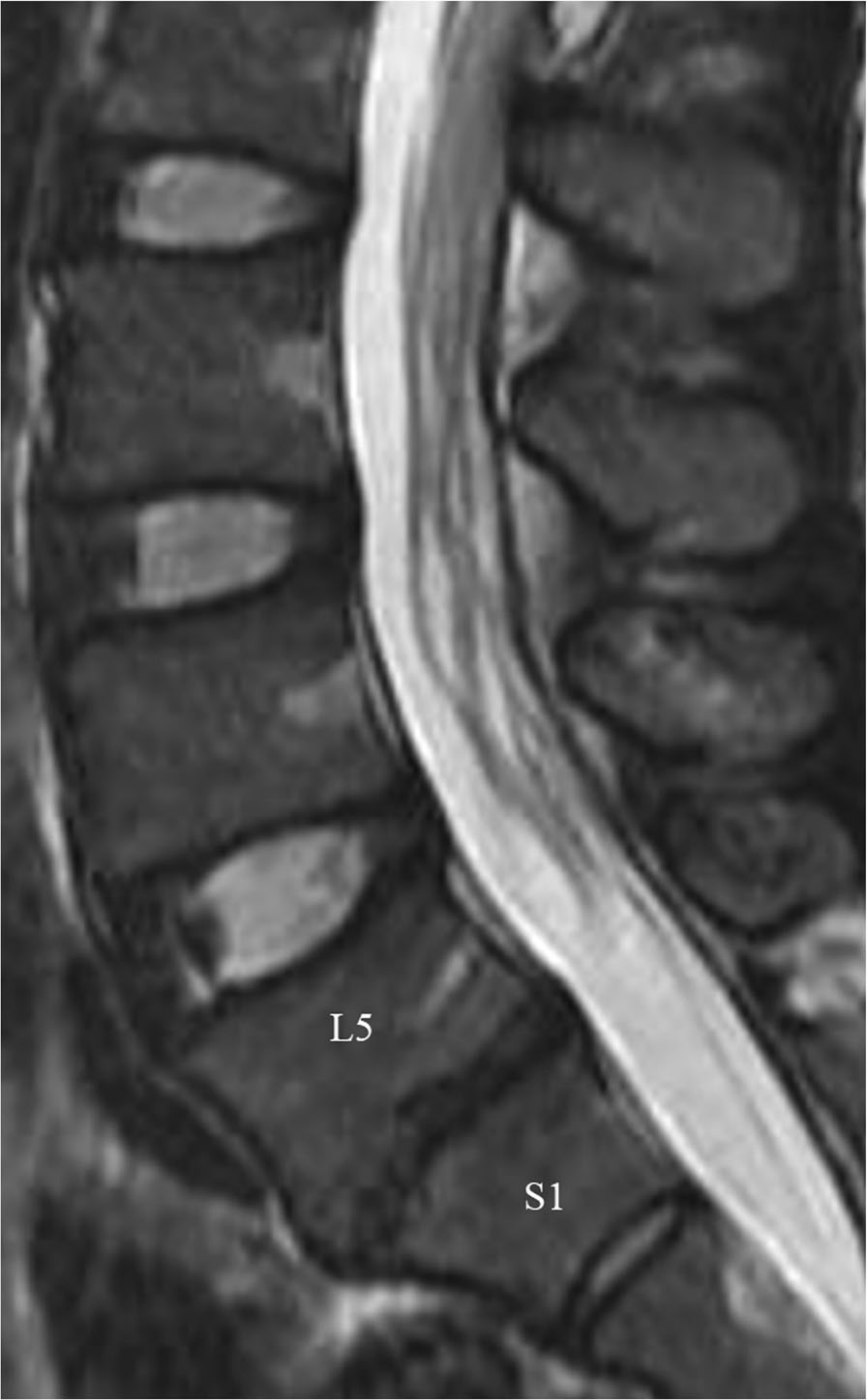
Sagittal T2 weighted images of the lumbar spine of a female athletics. There is disc dissecation and loss of disc height at L5-S1 disc with no distinction between the nucleus and annulus. Findings are consistent with Pfirrmann grade V. There are also associated endplate changes and marginal osteophytes at the same level

There were 4% or 15/406 disks with grade V or severe DDD (Figs. 4 and 5). L5-S1 levels were the most affected (47% or 7/15) followed by L3-L4 level (20% or 3/15). Females were more commonly affected than males (60% or 9/15 versus 40% or 6/15). Most of the affected discs even more so in Olympic athletes were seen above age of 30 (60% or 9/15 versus 40% or 6/15 between age of 20 and 30). Athletics were the sports most affected by grade V severe disc disease (47% or 7/15) followed by Rowing, weightlifting and table-tennis (13% or 2/15 for each one) and weightlifting (10% or 4/36).

## Discussion

Data about the degenerative changes of the cervical spine in athletes and particularly Olympic athletes is fairly limited [6, 7, 17–21]. Our study is the first to assess the cervical DDD in Olympic athletes who obtained imaging. Nearly 58% of the cervical discs of the examined athletes demonstrate some degree of degeneration: 75% were classified as mild and 25% as moderate to severe. The percentage of DDD of the cervical spine in our study was significantly higher than other studies of symptomatic and asymptomatic non-athletes of the same age groups. For instance, Boden et al. conducted an MRI study of the cervical spine in 63 asymptomatic subjects and reported that the disc was degenerated or narrowed at one level or more in 25% of subjects who were less than forty years old [22]. In another study involving 497 asymptomatic volunteers, Matsumoto found disc degeneration was the most common observation, being present in 17% of discs of men and 12% of women in their twenties [23]. Suzuki et al. evaluated cervical disc degeneration on MRI in a large population of symptomatic consecutive patients reporting neck pain or radiculopathy with or without neurologic deficits. The prevalence of cervical disc degeneration at more than 1 level was 41.0% in patients in their 20s [24]. Siivola showed that 25% of the cervical discs were degenerated in young adults with or without neck or shoulder pain of which 83% were classified as slightly degenerated and 17% as moderately degenerated [25].

Cervical degenerative changes were predominantly seen in men and above 30 years of age in our study. Shooters and Judo athletes were the most affected by mild DDD in our study whereas athletics, boxers and swimmers were the athletes most affected by moderate DDD. DDD was seen at an earlier age group (between 20 and 30 years of age) in swimmers which may be secondary to the increased mechanical stress on the cervical spine sustained when swimming [26]. On the other hand, DDD was only seen above 30 years of age in Athletics and Boxing athletes. Most of the degenerative discs were seen at the C5-C6 and C6-C7 levels where the fulcrum for maximal cervical spine movement is located [27].

At the thoracic spine, only mild degenerative changes were observed (12%). These changes were noted in female gymnastics and divers between 20 and 30 years of age. No moderate or severe DDD was noted at the thoracic level which may be secondary to the stability of the thoracic spine by the thoracic cage and the reduced mechanical stress on the thoracic intervertebral discs when compared to the cervical and the lumbar spine [17, 28].

At the level of the lumbar spine, nearly 39% of the lumbar discs demonstrated some degree of degeneration with two thirds of them classified as mild and one third as moderate or severe degeneration. Although lumbar disc degeneration is a common imaging finding in asymptomatic and symptomatic young individuals, our study showed that Olympic athletes have higher prevalence of moderate to severe disc degeneration than non-athletes [14, 29–31]. Even with the wide variation related to sample sizes, different age ranges, different clinical presentation, and different criteria and classification of the degenerative changes, our results are in accordance with several prior studies that showed the higher rate of these degenerative changes of the lumbar spine in athletes [1, 2, 6, 32–35]. For instance, Ong et al. studied the degenerative changes in elite athletes with lower back pain at the Sydney 2000 Olympic Games and showed degenerative changes of 36% of the discs at the L5/S1 level which is comparable to our study [6].

L4–5 and L5-S1 were the most commonly affected levels. Athletics were the most common athletes affected by DDD, followed by weightlifting and diving, respectively. The higher rate of degenerative changes in these sports is believed to be secondary to the repetitive, strenuous, and intense training required by the athletes to compete in the Olympic Games. For instance, Athletics are consistently exposed to considerable axial loading, flexion, and rotation that stresses the lumbar spine. Divers are also exposed to repetitive axial compression forces to the top of the head that may be transmitted caudally to the lumbar spine. Weightlifters sustain an increased axial loading across the entire thoracolumbar spine associated with an increased loading during the repetitive flexion and extension bending movements [36, 37].

Furthermore, our study showed that women athletes demonstrated a tendency for a higher rate of DDD in the lumbar region in contrast of what is reported in non-athletes at the same age groups where young men were more susceptible to disc degeneration than young women [29, 38, 39]. .This is likely related to the excessive mechanical stress and physical injury sustained by young elite women athletes.

In contrast of what our study noticed in the cervical spine where the degenerative discs were more frequently noted in athletes above 30 years of age, nearly 40–50% of the degenerative lumbar discs were noticed at an earlier age (i.e less than 30). This may be due to the fact the lumbar spine is the recipient of the heaviest biomechanical stress and is likely to be involved by degenerative disease earlier than the cervical spine.

Our results should be interpreted with caution and in the context of the patient’s clinical condition since more than one third of normal healthy subjects aged 21–30 years had degenerative discs in one study [40]. Furthermore, MRI-detectable abnormalities associated poorly with back pain in high-performing athletes [41].

The present study has several limitations. The descriptive and the retrospective nature of our study and the absence of detailed clinical correlation of the participant athletes are the main drawbacks. There is also a selection bias since the participants included in our study were not randomly selected and do not represent all the athletes. There may also be high prevalence of mild to moderate DDD in asymptomatic athletes which may not be detected because not all athletes had MR imaging. In addition, being a descriptive study, statistical significance was not calculated. Pfirrmann classification used in our study does not cover other morphological changes of the degenerative disease like endplate changes, osteophytes formation, disc protrusion or herniation, foraminal or spinal narrowing. Its extrapolation to the cervical and thoracic spine is also considered a shortcoming of our study.

## Conclusion

This is the first study to provide MRI mapping of degenerative disc disease of the spine in athletes competing at an elite level. The clinical significance of our paper is demonstrating that Olympic athletes have higher rates of moderate to severe degenerative disc disease of the cervical and lumbar spine than non-athletes which may expose them to higher risk of long-term sequelae of early DDD such as pain, instability, and neurologic damage. Athletes and coaches should aware of these results. Safe techniques and developing preventive strategies to protect the spine is of utmost importance.

## Supplementary information

**Additional file 1: Table S1.** Distribution of Pfirmanns type I-V cervical degenerative disc disease in athletes less than 20, 20–29 and > 30 years old by sport

**Additional file 2: Table S2.** Distribution of Pfirmanns type I-V cervical degenerative disc disease in Male and Female athletes by sport

## Abbreviations

C: Cervical, T thoracic
DDD: Degenerative disc disease
IOC: International Olympic committee
L: Lumbar
MRI: Magnetic resonance imaging

## References

[1] Swärd L, Hellstrom M, Jacobsson B, Pëterson L (1990). Back pain and radiologic changes in the thoraco-lumbar spine of athletes. Spine.

[2] Hangai M (2010). Relationship between low Back pain and competitive sports activities during youth. Am J Sports Med.

[3] Hangai M (2009). Lumbar Intervertebral Disk Degeneration in Athletes. Am J Sports Med.

[4] Bartolozzi C (1991). The incidence of disk changes in volleyball players. The magnetic resonance findings. Radiol Med.

[5] Kaneoka K (2007). Lumbar intervertebral disk degeneration in elite competitive swimmers: a case control study. Am J Sports Med.

[6] Ong A (2003). A pilot study of the prevalence of lumbar disc degeneration in elite athletes with lower back pain at the Sydney 2000 Olympic games. Br J Sports Med.

[7] Wasserman MS (2018). Evaluation of spine MRIs in athletes participating in the Rio de Janeiro 2016 Summer Olympic games. BMJ Open Sport Exerc Med.

[8] Goldstein JD, Berger PE, Windler GE, Jackson DW (1991). Spine injuries in gymnasts and swimmers. An epidemiologic investigation. Am J Sports Med.

[9] Baranto A, Hellström M, Cederlund C-G, Nyman R, Swärd L (2009). Back pain and MRI changes in the thoraco-lumbar spine of top athletes in four different sports: a 15-year follow-up study. Knee Surg Sports Traumatol Arthrosc.

[10] Kujala UM, Taimela S, Erkintalo M, Salminen JJ, Kaprio J (1996). Low-back pain in adolescent athletes. Med Sci Sports Exerc.

[11] Baranto A, Hellström M, Nyman R, Lundin O, Swärd L (2006). Back pain and degenerative abnormalities in the spine of young elite divers: a 5-year follow-up magnetic resonance imaging study. Knee Surg Sports Traumatol Arthrosc.

[12] T​horeson O, et al. Back pain and MRI abnormalities in the thoraco-lumbar spine of elite long distance runners. A cross sectional study. Medical Research Archives. [S.l.]. 2015;2(4). ISSN 2375-1924.

[13] Witwit WA (2018). Disc degeneration on MRI is more prevalent in young elite skiers compared to controls. Knee Surg Sports Traumatol Arthrosc.

[14] Teraguchi M (2014). Prevalence and distribution of intervertebral disc degeneration over the entire spine in a population-based cohort: the Wakayama spine study. Osteoarthr Cartil.

[15] Pfirrmann CW, Metzdorf A, Zanetti M, Hodler J, Boos N (2001). Magnetic resonance classification of lumbar intervertebral disc degeneration. Spine.

[16] Guermazi A (2018). Sports injuries at the Rio de Janeiro 2016 summer Olympics: use of diagnostic imaging services. Radiology.

[17] Daniels DJ (2015). Degenerative changes in adolescent spines: a comparison of motocross racers and age-matched controls. J Neurosurg Pediatr.

[18] Scher AT (1990). Premature onset of degenerative disease of the cervical spine in rugby players. S Afr Med J.

[19] Triantafillou KM, Lauerman W, Kalantar SB (2012). Degenerative disease of the cervical spine and its relationship to athletes. Clin Sports Med.

[20] O’Brien CP (1996). ‘Rugby neck’: cervical degeneration in two front row rugby union players. Clin J Sport Med.

[21] Kartal A, Yildiran I, Senköylü A, Korkusuz F (2004). Soccer causes degenerative changes in the cervical spine. Eur Spine J.

[22] Boden SD (1990). Abnormal magnetic-resonance scans of the cervical spine in asymptomatic subjects. A prospective investigation. J Bone Joint Surg Am.

[23] Matsumoto M (1998). MRI of cervical intervertebral discs in asymptomatic subjects. J Bone Joint Surg Br.

[24] Suzuki A (2018). Patterns of cervical disc degeneration: analysis of magnetic resonance imaging of over 1000 symptomatic subjects. Global Spine Journal.

[25] Siivola SM (2002). MRI changes of cervical spine in asymptomatic and symptomatic young adults. Eur Spine J.

[26] Guth EH (1995). A comparison of cervical rotation in age-matched adolescent competitive swimmers and healthy males. J Orthop Sports Phys Ther.

[27] Anderst WJ, Donaldson WF, Lee JY, Kang JD (2013). Cervical motion segment percent contributions to flexion-extension during continuous functional movement in control subjects and arthrodesis patients. Spine.

[28] McInerney J, Ball PA (2000). The pathophysiology of thoracic disc disease. Neurosurg Focus.

[29] Takatalo J (2009). Prevalence of degenerative imaging findings in lumbar magnetic resonance imaging among young adults. Spine.

[30] Brinjikji W (2015). Systematic literature review of imaging features of spinal degeneration in asymptomatic populations. Am J Neuroradiol.

[31] Romeo V (2019). High prevalence of spinal magnetic resonance imaging findings in asymptomatic young adults (18–22 Yrs) candidate to air force flight. SPINE.

[32] Dimitriadis AT (2011). Intervertebral disc changes after 1 h of running: a study on athletes. J Int Med Res.

[33] Burnett AF (1996). Thoracolumbar disc degeneration in young fast bowlers in cricket: a follow-up study. Clin Biomech (Bristol, Avon).

[34] Lundin O, Hellström M, Nilsson I, Swärd L (2001). Back pain and radiological changes in the thoraco-lumbar spine of athletes. A long-term follow-up. Scand J Med Sci Sports.

[35] Külling FA (2014). High prevalence of disc degeneration and Spondylolysis in the lumbar spine of Professional Beach volleyball players. Orthop J Sports Med.

[36] Vadalà G (2014). Early intervertebral disc degeneration changes in asymptomatic weightlifters assessed by T1ρ-magnetic resonance imaging. Spine.

[37] Aggrawal ND, Kaur R, Kumar S, Mathur DN (1979). A study of changes in the spine in weight lifters and other athletes. Br J Sports Med.

[38] Miller JA, Schmatz C, Schultz AB (1988). Lumbar disc degeneration: correlation with age, sex, and spine level in 600 autopsy specimens. Spine.

[39] Łebkowski WJ (2002). Ageing and degeneration of human lumbar intervertebral discs. Pol. Merkur Lekarski.

[40] Powell MC, Wilson M, Szypryt P, Symonds EM, Worthington BS (1986). Prevalence of lumbar disc degeneration observed by magnetic resonance in symptomless women. Lancet.

[41] Thoreson O (2017). The effect of repetitive flexion and extension fatigue loading on the young porcine lumbar spine, a feasibility study of MRI and histological analyses. J Exp Orthop.

