# Supplementary material for: MRI-detected spinal disc degenerative changes in athletes participating in the Rio de Janeiro 2016 Summer Olympics games
Source: BMC Musculoskelet Disord. 2020 Jan 20;21:45. doi: 10.1186/s12891-020-3057-3 (PMC6972034; doi:10.1186/s12891-020-3057-3)
Supplement: Supplementary file 1 — Additional file 1: Table S1. Distribution of Pfirmanns type I-V cervical degenerative disc disease in athletes less than 20, 20–29 and > 30 years old by sport [file 12891_2020_3057_MOESM1_ESM.docx]

**Additional file 1: Table S1.** Distribution of Pfirmanns type I-V cervical degenerative disc disease in athletes less than 20, 20-29 and >30 years old by sport

| **Sport** | **I** | | | **II** | | | **III** | | | **IV** | | | **V** | | |
| --- | --- | --- | --- | --- | --- | --- | --- | --- | --- | --- | --- | --- | --- | --- | --- |
|  |  |  |  |  |  |  |  |  |  |  |  |  |  |  |  |
| **Age** | **<20** | **20-29** | ≥**30** | **<20** | **20-29** | ≥**30** | **<20** | **20-29** | ≥**30** | **<20** | **20-29** | ≥**30** | **<20** | **20-29** | ≥**30** |
| Aquatics - Diving | 0 | 6 | 0 | 0 | 0 | 0 | 0 | 0 | 0 | 0 | 0 | 0 | 0 | 0 | 0 |
| Aquatics - Swimming | 0 | 1 | 0 | 0 | 0 | 0 | 0 | 2 | 0 | 0 | 3 | 0 | 0 | 0 | 0 |
| Aquatics - Water polo | 0 | 0 | 2 | 0 | 0 | 1 | 0 | 0 | 3 | 0 | 0 | 0 | 0 | 0 | 0 |
| Archery | 0 | 0 | 0 | 0 | 0 | 0 | 0 | 0 | 0 | 0 | 0 | 0 | 0 | 0 | 0 |
| Athletics | 0 | 0 | 1 | 0 | 0 | 0 | 0 | 0 | 0 | 0 | 0 | 5 | 0 | 0 | 0 |
| Badminton | 0 | 0 | 0 | 0 | 0 | 0 | 0 | 0 | 0 | 0 | 0 | 0 | 0 | 0 | 0 |
| Beach volleyball | 0 | 0 | 0 | 0 | 0 | 0 | 0 | 0 | 0 | 0 | 0 | 0 | 0 | 0 | 0 |
| Boxing | 0 | 0 | 1 | 0 | 0 | 0 | 0 | 0 | 0 | 0 | 0 | 5 | 0 | 0 | 0 |
| Canoe - Sprint | 0 | 0 | 1 | 0 | 0 | 0 | 0 | 0 | 5 | 0 | 0 | 0 | 0 | 0 | 0 |
| Cycling - MTB | 0 | 0 | 0 | 0 | 0 | 0 | 0 | 0 | 0 | 0 | 0 | 0 | 0 | 0 | 0 |
| Equestrian | 0 | 0 | 0 | 0 | 0 | 0 | 0 | 0 | 0 | 0 | 0 | 0 | 0 | 0 | 0 |
| Field Hockey | 0 | 0 | 3 | 0 | 0 | 3 | 0 | 0 | 0 | 0 | 0 | 0 | 0 | 0 | 0 |
| Fencing | 0 | 0 | 6 | 0 | 0 | 0 | 0 | 0 | 0 | 0 | 0 | 0 | 0 | 0 | 0 |
| Football | 0 | 1 | 0 | 0 | 5 | 0 | 0 | 0 | 0 | 0 | 0 | 0 | 0 | 0 | 0 |
| Gymnastics | 0 | 6 | 0 | 0 | 0 | 0 | 0 | 4 | 0 | 0 | 2 | 0 | 0 | 0 | 0 |
| Handball | 0 | 0 | 0 | 0 | 0 | 0 | 0 | 0 | 0 | 0 | 0 | 0 | 0 | 0 | 0 |
| Hockey | 0 | 0 | 3 | 0 | 0 | 0 | 0 | 0 | 3 | 0 | 0 | 0 | 0 | 0 | 0 |
| Judo | 0 | 6 | 3 | 0 | 0 | 0 | 0 | 6 | 3 | 0 | 0 | 0 | 0 | 0 | 0 |
| Rowing | 0 | 0 | 0 | 0 | 0 | 0 | 0 | 0 | 0 | 0 | 0 | 0 | 0 | 0 | 0 |
| Rugby | 0 | 0 | 0 | 0 | 0 | 0 | 0 | 0 | 0 | 0 | 0 | 0 | 0 | 0 | 0 |
| Sailing | 0 | 0 | 4 | 0 | 0 | 0 | 0 | 0 | 1 | 0 | 0 | 0 | 0 | 0 | 1 |
| Shooting | 0 | 0 | 2 | 0 | 0 | 0 | 0 | 0 | 10 | 0 | 0 | 0 | 0 | 0 | 0 |
| Table tennis | 0 | 0 | 0 | 0 | 0 | 0 | 0 | 0 | 0 | 0 | 0 | 0 | 0 | 0 | 0 |
| Tennis | 0 | 2 | 0 | 0 | 0 | 0 | 0 | 2 | 0 | 0 | 2 | 0 | 0 | 0 | 0 |
| Triathlon | 0 | 0 | 0 | 0 | 0 | 0 | 0 | 0 | 0 | 0 | 0 | 0 | 0 | 0 | 0 |
| Volleyball | 0 | 0 | 0 | 0 | 0 | 0 | 0 | 0 | 0 | 0 | 0 | 0 | 0 | 0 | 0 |
| Weightlifting | 0 | 0 | 0 | 0 | 0 | 0 | 0 | 0 | 0 | 0 | 0 | 0 | 0 | 0 | 0 |
| Wrestling | 0 | 1 | 4 | 0 | 2 | 0 | 0 | 3 | 2 | 0 | 0 | 0 | 0 | 0 | 0 |
| Total | 0 | 23 | 30 | 0 | 7 | 4 | 0 | 17 | 27 | 0 | 7 | 10 | 0 | 0 | 1 |

**Table 3b:** Distribution of Pfirmanns type I-V lumbar degenerative disc disease in athletes less than 20, 20-29 and >30 years old by sport

| **Sport** | **I** | | | **II** | | | **III** | | | **IV** | | | **V** | | |
| --- | --- | --- | --- | --- | --- | --- | --- | --- | --- | --- | --- | --- | --- | --- | --- |
|  |  |  |  |  |  |  |  |  |  |  |  |  |  |  |  |
| **Age** | **<20** | **20-29** | ≥**30** | **<20** | **20-29** | ≥**30** | **<20** | **20-29** | ≥**30** | **<20** | **20-29** | ≥**30** | **<20** | **20-29** | ≥**30** |
| Aquatics - Diving | 0 | 8 | 0 | 0 | 2 | 2 | 0 | 2 | 1 | 0 | 3 | 2 | 0 | 0 | 0 |
| Aquatics - Swimming | 0 | 8 | 0 | 5 | 0 | 0 | 0 | 2 | 0 | 0 | 0 | 0 | 0 | 0 | 0 |
| Aquatics - Water polo | 0 | 0 | 0 | 0 | 0 | 0 | 0 | 0 | 0 | 0 | 0 | 0 | 0 | 0 | 0 |
| Archery | 0 | 0 | 0 | 0 | 4 | 0 | 0 | 0 | 0 | 0 | 1 | 0 | 0 | 0 | 0 |
| Athletics | 10 | 67 | 29 | 0 | 14 | 5 | 0 | 6 | 5 | 0 | 8 | 4 | 0 | 5 | 2 |
| Badminton | 0 | 2 | 0 | 0 | 0 | 0 | 0 | 3 | 0 | 0 | 0 | 0 | 0 | 0 | 0 |
| Beach volleyball | 0 | 5 | 0 | 0 | 0 | 0 | 0 | 0 | 0 | 0 | 0 | 0 | 0 | 0 | 0 |
| Boxing | 0 | 8 | 10 | 0 | 0 | 0 | 0 | 2 | 0 | 0 | 0 | 0 | 0 | 0 | 0 |
| Canoe - Sprint | 0 | 0 | 0 | 0 | 0 | 0 | 0 | 0 | 0 | 0 | 0 | 0 | 0 | 0 | 0 |
| Cycling | 0 | 5 | 0 | 0 | 0 | 0 | 0 | 0 | 0 | 0 | 0 | 0 | 0 | 0 | 0 |
| Equestrian | 0 | 5 | 0 | 0 | 0 | 0 | 0 | 0 | 0 | 0 | 0 | 0 | 0 | 0 | 0 |
| Field Hockey | 0 | 0 | 0 | 0 | 0 | 0 | 0 | 0 | 0 | 0 | 0 | 0 | 0 | 0 | 0 |
| Fencing | 0 | 0 | 1 | 0 | 0 | 0 | 0 | 0 | 1 | 0 | 0 | 3 | 0 | 0 | 0 |
| Football | 0 | 4 | 0 | 0 | 0 | 0 | 0 | 1 | 0 | 0 | 0 | 0 | 0 | 0 | 0 |
| Gymnastics | 13 | 4 | 0 | 0 | 0 | 0 | 2 | 1 | 0 | 0 | 0 | 0 | 0 | 0 | 0 |
| Handball | 0 | 0 | 4 | 0 | 0 | 4 | 0 | 0 | 1 | 0 | 0 | 1 | 0 | 0 | 0 |
| Hockey | 0 | 3 | 0 | 0 | 0 | 0 | 0 | 0 | 0 | 0 | 2 | 0 | 0 | 0 | 0 |
| Judo | 0 | 9 | 8 | 0 | 5 | 3 | 0 | 0 | 2 | 0 | 1 | 1 | 0 | 0 | 1 |
| Rowing | 0 | 10 | 2 | 0 | 0 | 0 | 0 | 0 | 0 | 0 | 0 | 1 | 0 | 0 | 2 |
| Rugby | 0 | 0 | 4 | 0 | 0 | 0 | 0 | 0 | 0 | 0 | 0 | 1 | 0 | 0 | 0 |
| Sailing | 0 | 0 | 0 | 0 | 3 | 0 | 0 | 0 | 0 | 0 | 1 | 0 | 0 | 1 | 0 |
| Shooting | 0 | 0 | 1 | 0 | 0 | 6 | 0 | 0 | 2 | 0 | 0 | 1 | 0 | 0 | 0 |
| Table tennis | 0 | 0 | 0 | 0 | 0 | 3 | 0 | 0 | 0 | 0 | 0 | 0 | 0 | 0 | 2 |
| Tennis | 0 | 0 | 0 | 0 | 4 | 0 | 0 | 0 | 0 | 0 | 1 | 0 | 0 | 0 | 0 |
| Triathlon | 0 | 0 | 0 | 0 | 0 | 2 | 0 | 0 | 0 | 0 | 0 | 3 | 0 | 0 | 0 |
| Volleyball | 0 | 10 | 3 | 0 | 0 | 2 | 0 | 0 | 0 | 0 | 0 | 0 | 0 | 0 | 0 |
| Weightlifting | 7 | 3 | 4 | 0 | 2 | 3 | 2 | 3 | 0 | 1 | 2 | 1 | 0 | 0 | 2 |
| Wrestling | 0 | 0 | 0 | 0 | 0 | 0 | 0 | 0 | 0 | 0 | 0 | 0 | 0 | 0 | 0 |
| Total | 30 | 151 | 66 | 5 | 34 | 30 | 4 | 20 | 12 | 1 | 19 | 18 | 0 | 6 | 9 |
