# Supplementary material for: MRI-detected spinal disc degenerative changes in athletes participating in the Rio de Janeiro 2016 Summer Olympics games
Source: BMC Musculoskelet Disord. 2020 Jan 20;21:45. doi: 10.1186/s12891-020-3057-3 (PMC6972034; doi:10.1186/s12891-020-3057-3)
Supplement: Supplementary file 2 — Additional file 2: Table S2. Distribution of Pfirmanns type I-V cervical degenerative disc disease in Male and Female athletes by sport [file 12891_2020_3057_MOESM2_ESM.docx]

**Additional file 2: Table S2.** Distribution of Pfirmanns type I-V cervical degenerative disc disease in Male and Female athletes by sport

| **Sport** | **I** | | **II** | | **III** | | **IV** | | **V** | |
| --- | --- | --- | --- | --- | --- | --- | --- | --- | --- | --- |
| **Age** | **Female** | **Male** | **Female** | **Male** | **Female** | **Male** | **Female** | **Male** | **Female** | **Male** |
| Aquatics - Diving | 6 | 0 | 0 | 0 | 0 | 0 | 0 | 0 | 0 | 0 |
| Aquatics - Swimming | 0 | 1 | 0 | 0 | 0 | 2 | 0 | 3 | 0 | 0 |
| Aquatics - Water polo | 0 | 2 | 0 | 1 | 0 | 3 | 0 | 0 | 0 | 0 |
| Archery | 0 | 0 | 0 | 0 | 0 | 0 | 0 | 0 | 0 | 0 |
| Athletics | 1 | 0 | 0 | 0 | 0 | 0 | 5 | 0 | 0 | 0 |
| Badminton | 0 | 0 | 0 | 0 | 0 | 0 | 0 | 0 | 0 | 0 |
| Beach volleyball | 0 | 0 | 0 | 0 | 0 | 0 | 0 | 0 | 0 | 0 |
| Boxing | 0 | 1 | 0 | 0 | 0 | 0 | 0 | 5 | 0 | 0 |
| Canoe - Sprint | 0 | 1 | 0 | 0 | 0 | 5 | 0 | 0 | 0 | 0 |
| Cycling | 0 | 0 | 0 | 0 | 0 | 0 | 0 | 0 | 0 | 0 |
| Equestrian | 0 | 0 | 0 | 0 | 0 | 0 | 0 | 0 | 0 | 0 |
| Field Hockey | 0 | 3 | 0 | 3 | 0 | 0 | 0 | 0 | 0 | 0 |
| Fencing | 6 | 0 | 0 | 0 | 0 | 0 | 0 | 0 | 0 | 0 |
| Football | 0 | 1 | 0 | 5 | 0 | 0 | 0 | 0 | 0 | 0 |
| Gymnastics | 5 | 1 | 0 | 0 | 1 | 3 | 0 | 2 | 0 | 0 |
| Handball | 0 | 0 | 0 | 0 | 0 | 0 | 0 | 0 | 0 | 0 |
| Hockey | 3 | 0 | 0 | 0 | 3 | 0 | 0 | 0 | 0 | 0 |
| Judo | 0 | 9 | 0 | 0 | 0 | 9 | 0 | 0 | 0 | 0 |
| Rowing | 0 | 0 | 0 | 0 | 0 | 0 | 0 | 0 | 0 | 0 |
| Rugby | 0 | 0 | 0 | 0 | 0 | 0 | 0 | 0 | 0 | 0 |
| Sailing | 0 | 4 | 0 | 0 | 0 | 1 | 0 | 0 | 0 | 1 |
| Shooting | 0 | 2 | 0 | 0 | 0 | 10 | 0 | 0 | 0 | 0 |
| Table tennis | 0 | 0 | 0 | 0 | 0 | 0 | 0 | 0 | 0 | 0 |
| Tennis | 0 | 2 | 0 | 0 | 0 | 2 | 0 | 2 | 0 | 0 |
| Triathlon | 0 | 0 | 0 | 0 | 0 | 0 | 0 | 0 | 0 | 0 |
| Volleyball | 0 | 0 | 0 | 0 | 0 | 0 | 0 | 0 | 0 | 0 |
| Weightlifting | 0 | 0 | 0 | 0 | 0 | 0 | 0 | 0 | 0 | 0 |
| Wrestling | 0 | 5 | 0 | 2 | 0 | 5 | 0 | 0 | 0 | 0 |
| Total | 21 | 32 | 0 | 11 | 4 | 40 | 5 | 12 | 0 | 1 |

**Table 4b:** Distribution of Pfirmanns type I-V lumbar degenerative disc disease in Male and Female athletes by sport

| **Sport** | **I** | | **II** | | **III** | | **IV** | | **V** | |
| --- | --- | --- | --- | --- | --- | --- | --- | --- | --- | --- |
| **Age** | **Female** | **Male** | **Female** | **Male** | **Female** | **Male** | **Female** | **Male** | **Female** | **Male** |
| Aquatics - Diving | 5 | 3 | 2 | 2 | 2 | 1 | 1 | 4 | 0 | 0 |
| Aquatics - Swimming | 4 | 4 | 5 | 0 | 1 | 1 | 0 | 0 | 0 | 0 |
| Aquatics - Water polo | 0 | 0 | 0 | 0 | 0 | 0 | 0 | 0 | 0 | 0 |
| Archery | 0 | 0 | 0 | 4 | 0 | 0 | 0 | 1 | 0 | 0 |
| Athletics | 73 | 33 | 9 | 10 | 10 | 1 | 9 | 3 | 4 | 3 |
| Badminton | 0 | 2 | 0 | 0 | 0 | 3 | 0 | 0 | 0 | 0 |
| Beach volleyball | 5 | 0 | 0 | 0 | 0 | 0 | 0 | 0 | 0 | 0 |
| Boxing | 5 | 13 | 0 | 0 | 0 | 2 | 0 | 0 | 0 | 0 |
| Canoe - Sprint | 0 | 0 | 0 | 0 | 0 | 0 | 0 | 0 | 0 | 0 |
| Cycling - MTB | 5 | 0 | 0 | 0 | 0 | 0 | 0 | 0 | 0 | 0 |
| Equestrian | 0 | 5 | 0 | 0 | 0 | 0 | 0 | 0 | 0 | 0 |
| Field Hockey | 0 | 0 | 0 | 0 | 0 | 0 | 0 | 0 | 0 | 0 |
| Fencing | 0 | 1 | 0 | 0 | 0 | 1 | 0 | 3 | 0 | 0 |
| Football | 4 | 0 | 0 | 0 | 1 | 0 | 0 | 0 | 0 | 0 |
| Gymnastics | 17 | 0 | 0 | 0 | 3 | 0 | 0 | 0 | 0 | 0 |
| Handball | 0 | 4 | 4 | 0 | 1 | 0 | 0 | 1 | 0 | 0 |
| Hockey | 3 | 0 | 0 | 0 | 0 | 0 | 2 | 0 | 0 | 0 |
| Judo | 4 | 13 | 5 | 3 | 1 | 1 | 0 | 2 | 0 | 1 |
| Rowing | 12 | 0 | 0 | 0 | 0 | 0 | 1 | 0 | 2 | 0 |
| Rugby | 4 | 0 | 0 | 0 | 0 | 0 | 1 | 0 | 0 | 0 |
| Sailing | 0 | 0 | 3 | 0 | 0 | 0 | 1 | 0 | 1 | 0 |
| Shooting | 0 | 1 | 0 | 6 | 0 | 2 | 0 | 1 | 0 | 0 |
| Table tennis | 0 | 0 | 0 | 3 | 0 | 0 | 0 | 0 | 0 | 2 |
| Tennis | 0 | 0 | 0 | 4 | 0 | 0 | 0 | 1 | 0 | 0 |
| Triathlon | 0 | 0 | 2 | 0 | 0 | 0 | 3 | 0 | 0 | 0 |
| Volleyball | 13 | 0 | 2 | 0 | 0 | 0 | 0 | 0 | 0 | 0 |
| Weightlifting | 8 | 6 | 1 | 4 | 1 | 4 | 3 | 1 | 2 | 0 |
| Wrestling | 0 | 0 | 0 | 0 | 0 | 0 | 0 | 0 | 0 | 0 |
| Total | 162 | 85 | 33 | 36 | 20 | 16 | 21 | 17 | 9 | 6 |
